# Supplementary material for: The interactome of intact mitochondria by cross-linking mass spectrometry provides evidence for coexisting respiratory supercomplexes
Source: Mol Cell Proteomics. 2017 Dec 8;17(2):216–32. doi: 10.1074/mcp.RA117.000470 (PMC5795388; doi:10.1074/mcp.RA117.000470)
Supplement: Supplemental Data [file supp_RA117.000470_133922_0_supp_23978_fzffwf.zip › spectra_annotation/mito_spectra_annotation/108-1-6-1-6-1.pdf]

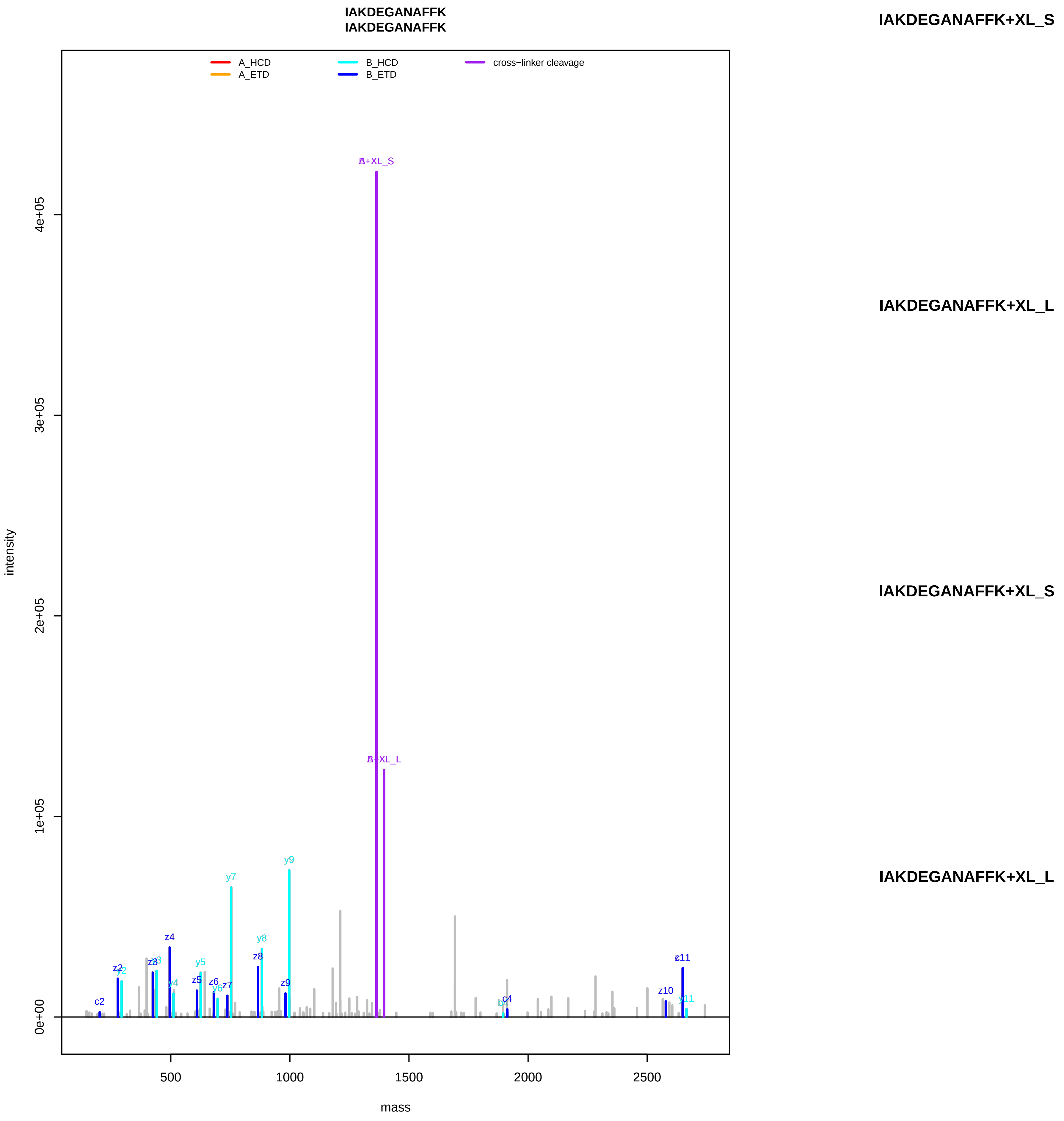
IAKDEGANAFFK
IAKDEGANAFFK
IAKDEGANAFFK+XL_S
IAKDEGANAFFK+XL_L
IAKDEGANAFFK+XL_S
IAKDEGANAFFK+XL_L
A_HCD
A_ETD
B_HCD
B_ETD
cross−linker cleavage
B+XL_S
B+XL_L
y9
y7
y8
z4
z2
y2
z3
y3
z8
y5
z5
z6
y6
z7
y4
z9
c2
c4
z10
c11
y11
intensity
0e+00
1e+05
2e+05
3e+05
4e+05
mass
500
1000
1500
2000
2500
